# New Insights into Personalized Surgical Oncology

**DOI:** 10.3390/jpm15070295

**Published:** 2025-07-08

**Authors:** Maximos Frountzas

**Affiliations:** First Propaedeutic Department of Surgery, National and Kapodistrian University of Athens, Hippocration General Hospital, 115 27 Athens, Greece; froumax@hotmail.com; Tel.: +30-6978880045

## 1. The Evolution of Surgical Oncology

The trajectory of surgical oncology has evolved dramatically during recent years, transitioning from widely applied treatment protocols to highly individualized therapeutic strategies according to each tumor’s specific characteristics. After the recent outburst of research in this field, personalized surgical oncology now merges data-rich molecular tumor profiling, real-time computational tools, and immune-based insights to guide tailored surgical decisions and achieve better treatment outcomes.

A cornerstone to this evolution is surgical research, which has been helped by circulating tumor DNA (ctDNA) sequencing advances. Genomic biomarker detection in advanced solid tumors could facilitate large-scale prospective trials by enabling faster screening duration and higher trial enrollment rates, as demonstrated in the SCRUM-Japan GI-SCREEN and GOZILA studies [1]. Moreover, the clonal architecture of ctDNA profiling offers the chance for more accurate targeting of oncogenic drivers and might improve study outcomes [2]. In addition, multiplex immunochemistry and immunofluorescence have emerged as potent tools for the simultaneous detection of multiple protein biomarkers in a single tissue section, thereby expanding opportunities for molecular and immune profiling while preserving tissue samples. This can provide unprecedented insights into the tumor microenvironment, including the potential interplay between various cell types and possible therapeutic targets [3].

The molecular analysis of surgical specimens can also be utilized for genomic profiling, as specific genetic alterations, such as epidermal growth factor receptor (EGFR), Kirsten rat sarcoma virus (KRAS), and phosphoinositide 3-kinase (PI3K) mutations, in resected lung specimens have been correlated with prognostic and therapeutic benefit [4]. Furthermore, the molecular residual disease (MRD) detection through ctDNA-based techniques seems to present promising outcomes regarding preoperative prognostic value in early-stage lung cancer [5] and postoperative adjuvant response in patients with colorectal cancer and colorectal liver metastatic disease [6,7]. These findings underscore the need to embed molecular diagnostics into surgical workflows.

## 2. Insights from This Special Issue

The contributions in this Special Issue, entitled “New Insights into Personalized Surgical Oncology”, further illuminate the multifaceted nature of personalized surgical oncology. Pergialiotis et al. explored the complex landscape of cervical cancer surgery, reviewing evidence for de-escalated procedures and the individualized use of sentinel lymph node mapping (Contribution 1). This research reinforces the principle that less radical interventions may suffice in carefully stratified early-stage cases, aligning with evolving guidelines. On the other hand, they highlighted the significance of multimodal treatment in the context of fertility sparing for selected patients and the role of prophylactic follow-up vaccination against oncogenic viruses, such as human papillomavirus (HPV).

In the context of colorectal cancer liver metastases (CRLMs), Račkauskas et al. examined the interplay between chemotherapy and liver regenerative capacity post-hepatectomy (Contribution 2). Patients with CRLMs presented reduced regenerative liver capacity compared to patients with benign indications. Moreover, lower serum transforming growth factor beta 1 (TGF-β1) levels and reduced TGF-β1 expression in liver tissue were associated with compromised liver regeneration, suggesting TGF-β1 involvement in mechanisms hindering liver regeneration capacity following major resection after chemotherapy. Rastrelli et al. investigated a synergistic approach to advanced in-transit melanoma using isolated limb hyperthermic–antiblastic perfusion (ILP) combined with immunotherapy (Contribution 3). Their findings supported a hybrid model where regional and systemic therapies converge, improving both overall survival and local disease-free survival. Nevertheless, they highlighted the need for future studies to investigate the optimal combination among locoregional and systemic therapies, as well as the time sequence of such modalities to achieve the highest local response rate.

Marazzi et al. shifted attention to progressive breast cancer after the induction of neoadjuvant systemic treatment in a confined number of lesions, which is termed “oligoprogression”, conducting a feasibility study utilizing radiotherapy in 50 patients (Contribution 4). The average progression-free survival (PFS) after radiotherapy was 13.7 months (95% CI 8.6–18.8 months), and only 18.6% of patients required a change in systemic treatment due to disease progression after six months, indicating that radiotherapy might play a role in prolonging PFS while enhancing disease control. Their analysis introduces a framework for integrating locoregional interventions within systemic regimens, particularly relevant in the context of personalized metastatic management, especially when conventional treatment approaches have been proven ineffective. Finally, Nibid et al. addressed a foundational element of precision surgery: molecular testing quality (Contribution 5). Their study on the optimization of RNA-sequencing samples highlighted that non-small cell lung cancer (NSCLC) samples with a high RNA concentration and fragmentation index and a low fixation and storage time, obtained from small biopsies, wedge resections, or metastasectomy, should be preferred for testing using RNA sequencing.

## 3. Future Perspectives of Surgical Oncology

The expanding frontiers of personalized surgical oncology offer a vision of malignancy management that is predictive, adaptive, and profoundly patient-centered. The growing capability to stratify risk and guide therapy using molecular and digital biomarkers is not only enhancing surgical precision—it is reshaping the fundamental approach to oncologic treatment. Meanwhile, artificial intelligence (AI) is revolutionizing predictive modeling, histologic assessment, and radiomics. AI models can predict chemotherapy response in metastatic colorectal cancer or gastric cancer [8,9], infer immune gene signatures from routine histology in hepatocellular carcinoma [10], and synthesize multimodal inputs for individualized treatment decisions using mechanistic modeling [11,12]. Finally, augmented and mixed reality technologies are transforming surgical planning, enabling the interactive 3D mapping of complex anatomical and oncologic structures, as well as enhancing margin visualization, especially in head and neck, renal, and musculoskeletal tumors [13,14,15]. Accessibility is a major issue in personalized surgical oncology, as most healthcare systems worldwide provide limited resources for such innovative means of cancer treatment. Nevertheless, the gap of infrastructure and expertise in low- and middle-income countries could be bridged by AI, improving equity in cancer care and allowing a large portion of patients to access such revolutionary services [16]. Together, these innovations redefine what is surgically possible and personalize surgically appropriate procedures.

From a therapeutic perspective, neoantigen-based vaccines are demonstrating real-world feasibility in pancreatic and glioblastoma trials, where de novo immune-mediated T-cell responses seem to delay or prevent tumor recurrence [17,18]. Such strategies may one day become integral to the postoperative management of selected tumor types. Ultimately, personalized surgical oncology is not defined by any single innovation but by the integration of many—genomics, imaging, immunology, and informatics. With the synthesis and application of these disciplines, we can move closer to truly individualized surgical care: one where biology guides the blade and precision defines the path.
